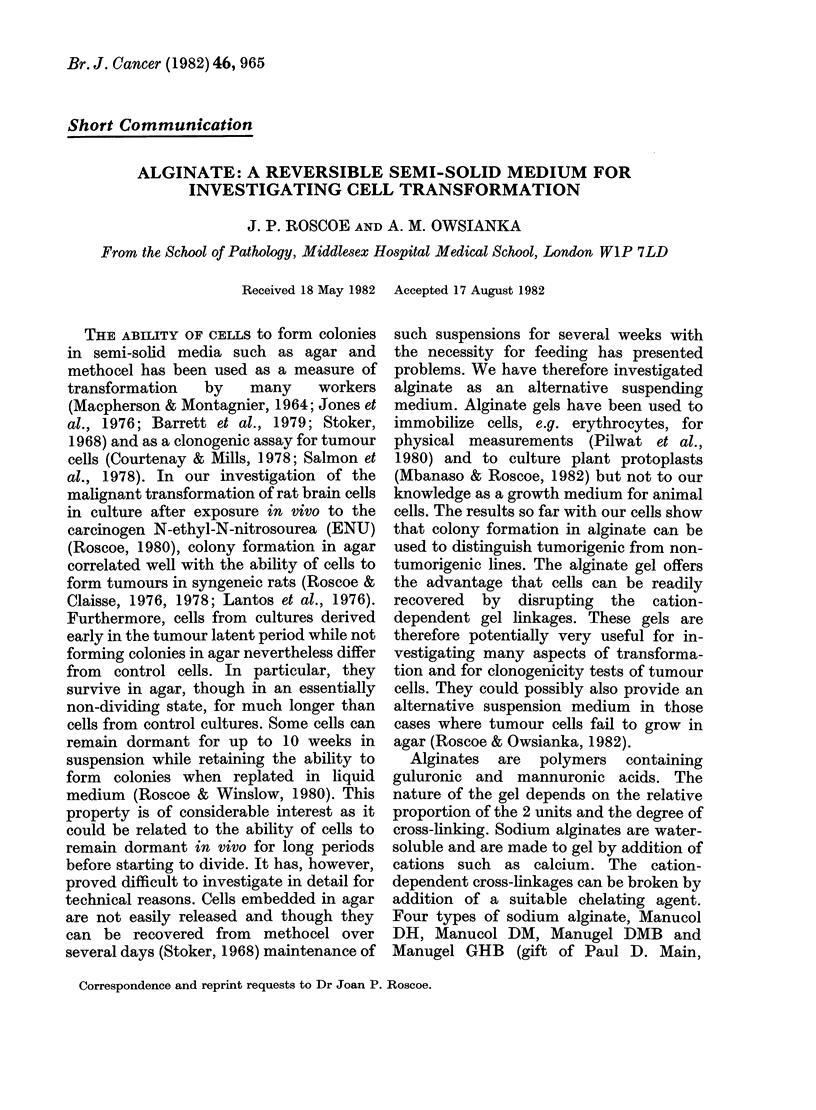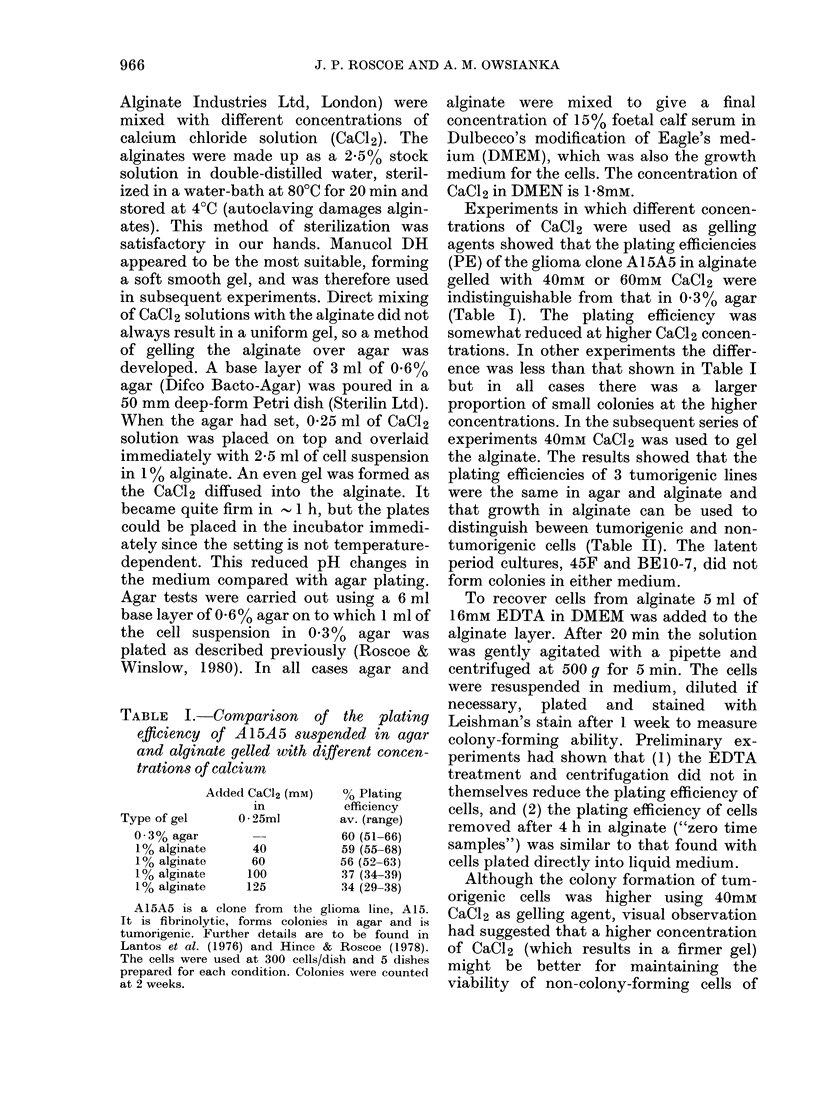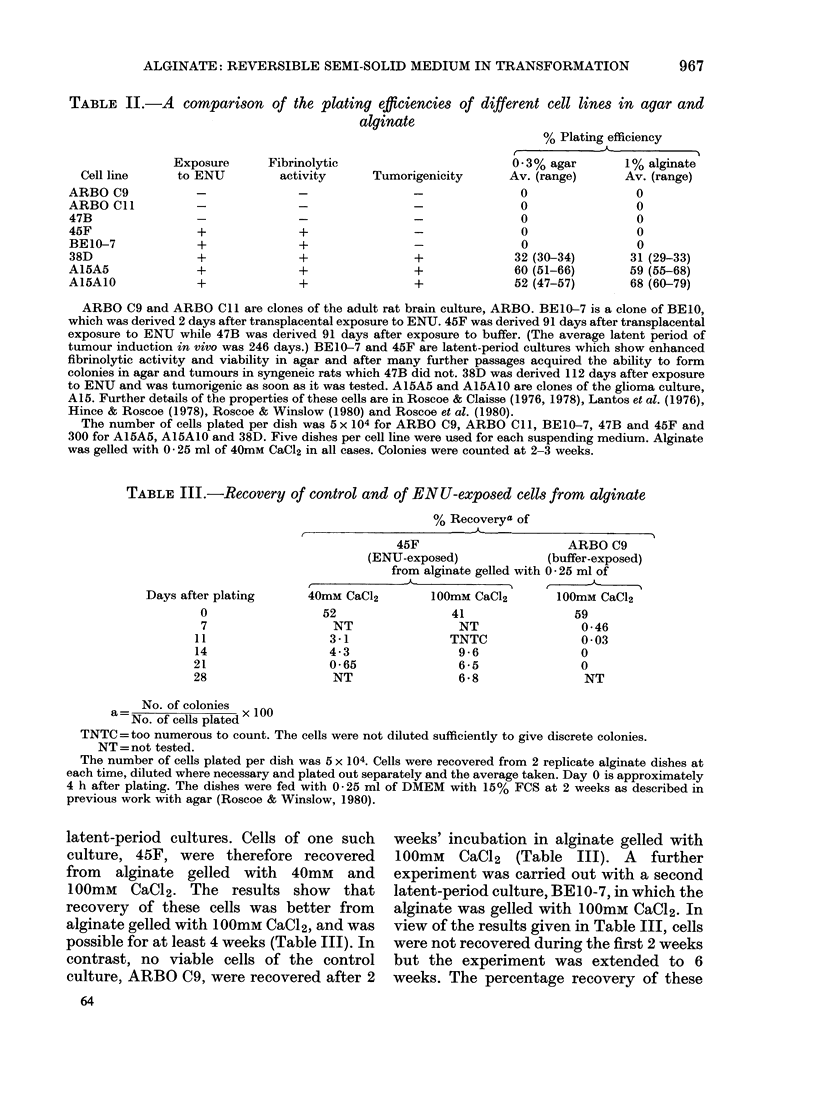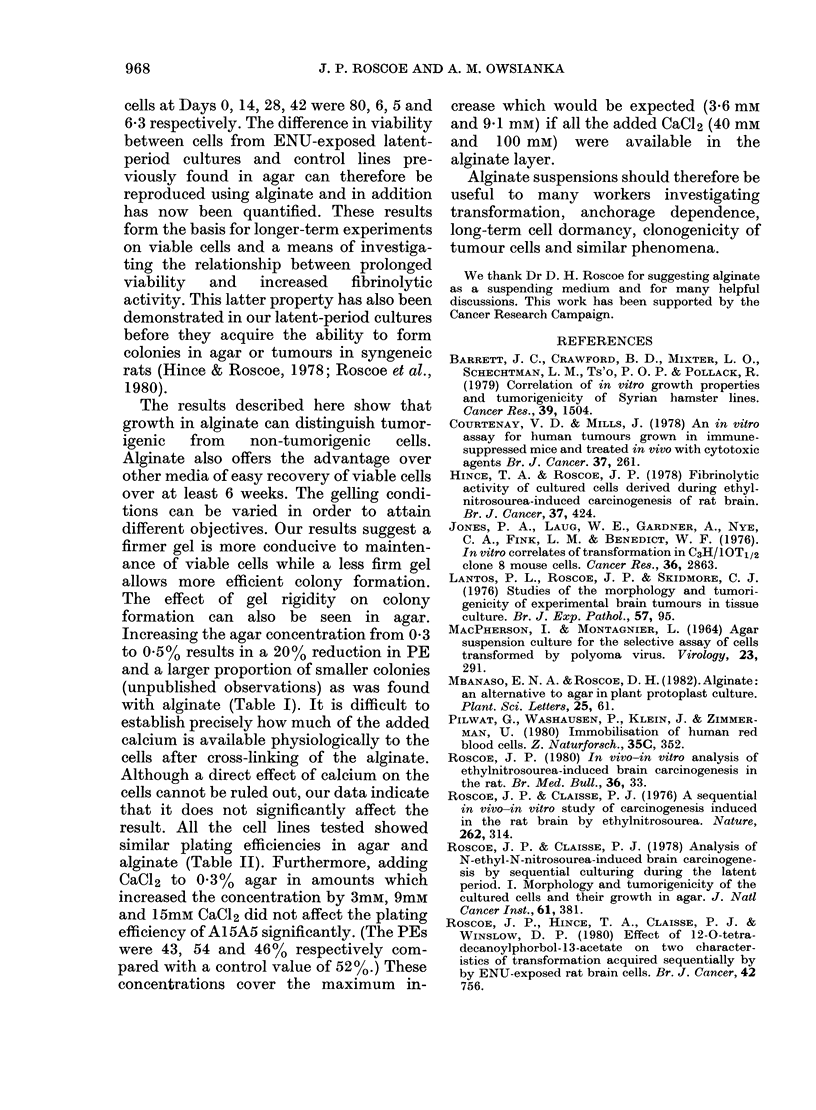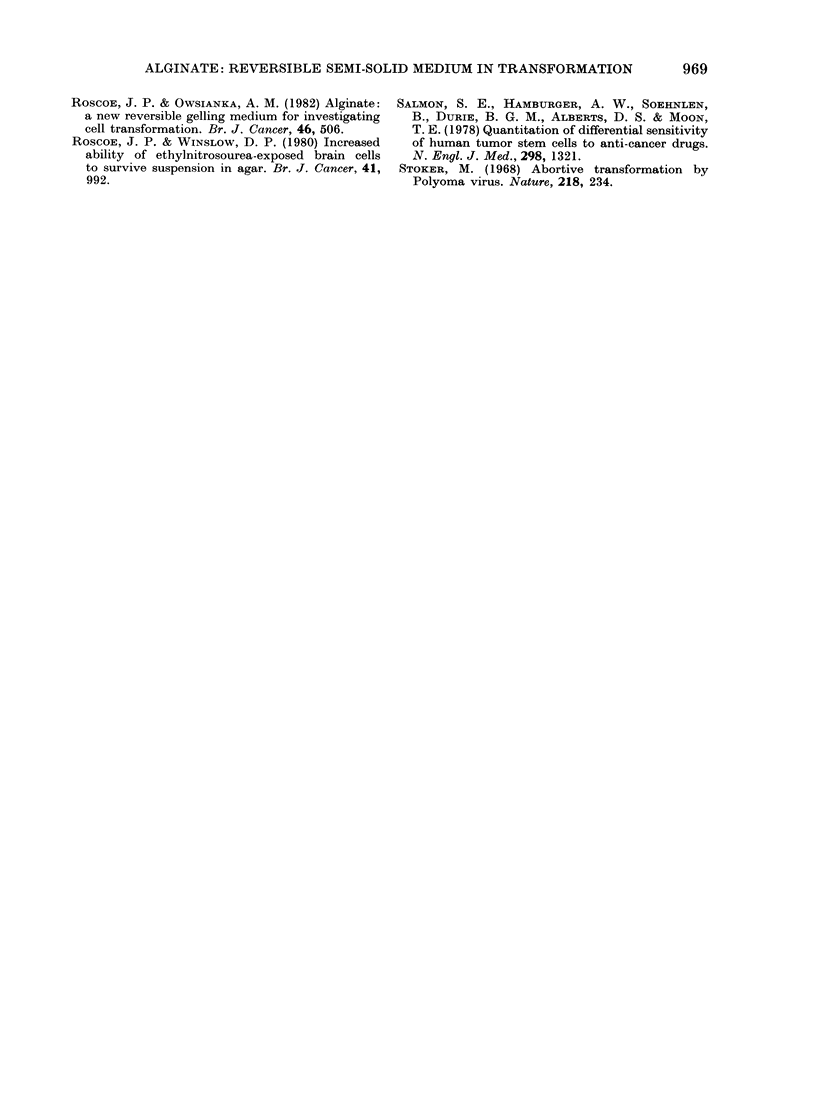# Alginate: a reversible semi-solid medium for investigating cell transformation.

**DOI:** 10.1038/bjc.1982.309

**Published:** 1982-12

**Authors:** J. P. Roscoe, A. M. Owsianka


*Br. J. Cancer* (1982) **46**, 965

***Short Communication***

### ALGINATE: A REVERSIBLE SEMI-SOLID MEDIUM FOR INVESTIGATING CELL TRANSFORMATION

J. P. ROSCOE AND A. M. OWSIANKA

*From the School of Pathology, Middlesex Hospital Medical School, London W1P 7LD*

Received 18 May 1982 Accepted 17 August 1982

THE ABILITY OF CELLS to form colonies in semi-solid media such as agar and methocel has been used as a measure of transformation by many workers (Macpherson & Montagnier, 1964; Jones *et al.*, 1976; Barrett *et al.*, 1979; Stoker, 1968) and as a clonogenic assay for tumour cells (Courtenay & Mills, 1978; Salmon *et al.*, 1978). In our investigation of the malignant transformation of rat brain cells in culture after exposure *in vivo* to the carcinogen N-ethyl-N-nitrosourea (ENU) (Roscoe, 1980), colony formation in agar correlated well with the ability of cells to form tumours in syngeneic rats (Roscoe & Claisse, 1976, 1978; Lantos *et al.*, 1976). Furthermore, cells from cultures derived early in the tumour latent period while not forming colonies in agar nevertheless differ from control cells. In particular, they survive in agar, though in an essentially non-dividing state, for much longer than cells from control cultures. Some cells can remain dormant for up to 10 weeks in suspension while retaining the ability to form colonies when replated in liquid medium (Roscoe & Winslow, 1980). This property is of considerable interest as it could be related to the ability of cells to remain dormant *in vivo* for long periods before starting to divide. It has, however, proved difficult to investigate in detail for technical reasons. Cells embedded in agar are not easily released and though they can be recovered from methocel over several days (Stoker, 1968) maintenance of such suspensions for several weeks with the necessity for feeding has presented problems. We have therefore investigated alginate as an alternative suspending medium. Alginate gels have been used to immobilize cells, *e.g.* erythrocytes, for physical measurements (Pilwat *et al.*, 1980) and to culture plant protoplasts (Mbanaso & Roscoe, 1982) but not to our knowledge as a growth medium for animal cells. The results so far with our cells show that colony formation in alginate can be used to distinguish tumorigenic from non-tumorigenic lines. The alginate gel offers the advantage that cells can be readily recovered by disrupting the cation-dependent gel linkages. These gels are therefore potentially very useful for investigating many aspects of transformation and for clonogenicity tests of tumour cells. They could possibly also provide an alternative suspension medium in those cases where tumour cells fail to grow in agar (Roscoe & Owsianka, 1982).

Alginates are polymers containing guluronic and mannuronic acids. The nature of the gel depends on the relative proportion of the 2 units and the degree of cross-linking. Sodium alginates are water-soluble and are made to gel by addition of cations such as calcium. The cation-dependent cross-linkages can be broken by addition of a suitable chelating agent. Four types of sodium alginate, Manucol DH, Manucol DM, Manugel DMB and Manugel GHB (gift of Paul D. Main,

Correspondence and reprint requests to Dr Joan P. Roscoe.

Alginate Industries Ltd, London) were mixed with different concentrations of calcium chloride solution ($CaCl_2$). The alginates were made up as a 2·5% stock solution in double-distilled water, sterilized in a water-bath at 80°C for 20 min and stored at 4°C (autoclaving damages alginates). This method of sterilization was satisfactory in our hands. Manucol DH appeared to be the most suitable, forming a soft smooth gel, and was therefore used in subsequent experiments. Direct mixing of $CaCl_2$ solutions with the alginate did not always result in a uniform gel, so a method of gelling the alginate over agar was developed. A base layer of 3 ml of 0·6% agar (Difco Bacto-Agar) was poured in a 50 mm deep-form Petri dish (Sterilin Ltd). When the agar had set, 0·25 ml of $CaCl_2$ solution was placed on top and overlaid immediately with 2·5 ml of cell suspension in 1% alginate. An even gel was formed as the $CaCl_2$ diffused into the alginate. It became quite firm in ~1 h, but the plates could be placed in the incubator immediately since the setting is not temperature-dependent. This reduced pH changes in the medium compared with agar plating. Agar tests were carried out using a 6 ml base layer of 0·6% agar on to which 1 ml of the cell suspension in 0·3% agar was plated as described previously (Roscoe & Winslow, 1980). In all cases agar and alginate were mixed to give a final concentration of 15% foetal calf serum in Dulbecco's modification of Eagle's medium (DMEM), which was also the growth medium for the cells. The concentration of $CaCl_2$ in DMEN is 1·8mM.

TABLE I.—*Comparison of the plating efficiency of A15A5 suspended in agar and alginate gelled with different concentrations of calcium*

| Type of gel | Added $CaCl_2$ (mM) in 0·25ml | % Plating efficiency av. (range) |
|---|---|---|
| 0·3% agar | — | 60 (51–66) |
| 1% alginate | 40 | 59 (55–68) |
| 1% alginate | 60 | 56 (52–63) |
| 1% alginate | 100 | 37 (34–39) |
| 1% alginate | 125 | 34 (29–38) |

A15A5 is a clone from the glioma line, A15. It is fibrinolytic, forms colonies in agar and is tumorigenic. Further details are to be found in Lantos *et al.* (1976) and Hince & Roscoe (1978). The cells were used at 300 cells/dish and 5 dishes prepared for each condition. Colonies were counted at 2 weeks.

Experiments in which different concentrations of $CaCl_2$ were used as gelling agents showed that the plating efficiencies (PE) of the glioma clone A15A5 in alginate gelled with 40mM or 60mM $CaCl_2$ were indistinguishable from that in 0·3% agar (Table I). The plating efficiency was somewhat reduced at higher $CaCl_2$ concentrations. In other experiments the difference was less than that shown in Table I but in all cases there was a larger proportion of small colonies at the higher concentrations. In the subsequent series of experiments 40mM $CaCl_2$ was used to gel the alginate. The results showed that the plating efficiencies of 3 tumorigenic lines were the same in agar and alginate and that growth in alginate can be used to distinguish beween tumorigenic and non-tumorigenic cells (Table II). The latent period cultures, 45F and BE10-7, did not form colonies in either medium.

To recover cells from alginate 5 ml of 16mM EDTA in DMEM was added to the alginate layer. After 20 min the solution was gently agitated with a pipette and centrifuged at 500 *g* for 5 min. The cells were resuspended in medium, diluted if necessary, plated and stained with Leishman's stain after 1 week to measure colony-forming ability. Preliminary experiments had shown that (1) the EDTA treatment and centrifugation did not in themselves reduce the plating efficiency of cells, and (2) the plating efficiency of cells removed after 4 h in alginate ("zero time samples") was similar to that found with cells plated directly into liquid medium.

Although the colony formation of tumorigenic cells was higher using 40mM $CaCl_2$ as gelling agent, visual observation had suggested that a higher concentration of $CaCl_2$ (which results in a firmer gel) might be better for maintaining the viability of non-colony-forming cells of

TABLE II.—*A comparison of the plating efficiencies of different cell lines in agar and alginate*

| Cell line | Exposure to ENU | Fibrinolytic activity | Tumorigenicity | % Plating efficiency: 0·3% agar Av. (range) | % Plating efficiency: 1% alginate Av. (range) |
|---|---|---|---|---|---|
| ARBO C9 | − | − | − | 0 | 0 |
| ARBO C11 | − | − | − | 0 | 0 |
| 47B | − | − | − | 0 | 0 |
| 45F | + | + | − | 0 | 0 |
| BE10–7 | + | + | − | 0 | 0 |
| 38D | + | + | + | 32 (30–34) | 31 (29–33) |
| A15A5 | + | + | + | 60 (51–66) | 59 (55–68) |
| A15A10 | + | + | + | 52 (47–57) | 68 (60–79) |

ARBO C9 and ARBO C11 are clones of the adult rat brain culture, ARBO. BE10–7 is a clone of BE10, which was derived 2 days after transplacental exposure to ENU. 45F was derived 91 days after transplacental exposure to ENU while 47B was derived 91 days after exposure to buffer. (The average latent period of tumour induction *in vivo* was 246 days.) BE10–7 and 45F are latent-period cultures which show enhanced fibrinolytic activity and viability in agar and after many further passages acquired the ability to form colonies in agar and tumours in syngeneic rats which 47B did not. 38D was derived 112 days after exposure to ENU and was tumorigenic as soon as it was tested. A15A5 and A15A10 are clones of the glioma culture, A15. Further details of the properties of these cells are in Roscoe & Claisse (1976, 1978), Lantos *et al.* (1976), Hince & Roscoe (1978), Roscoe & Winslow (1980) and Roscoe *et al.* (1980).

The number of cells plated per dish was $5 \times 10^4$ for ARBO C9, ARBO C11, BE10–7, 47B and 45F and 300 for A15A5, A15A10 and 38D. Five dishes per cell line were used for each suspending medium. Alginate was gelled with 0·25 ml of 40mM $CaCl_2$ in all cases. Colonies were counted at 2–3 weeks.

TABLE III.—*Recovery of control and of ENU-exposed cells from alginate*

| | % Recovery[a] of 45F (ENU-exposed) from alginate gelled with 0·25 ml of | | ARBO C9 (buffer-exposed) |
|---|---|---|---|
| Days after plating | 40mM $CaCl_2$ | 100mM $CaCl_2$ | 100mM $CaCl_2$ |
| 0 | 52 | 41 | 59 |
| 7 | NT | NT | 0·46 |
| 11 | 3·1 | TNTC | 0·03 |
| 14 | 4·3 | 9·6 | 0 |
| 21 | 0·65 | 6·5 | 0 |
| 28 | NT | 6·8 | NT |

$$a = \frac{\text{No. of colonies}}{\text{No. of cells plated}} \times 100$$

TNTC = too numerous to count. The cells were not diluted sufficiently to give discrete colonies.
NT = not tested.

The number of cells plated per dish was $5 \times 10^4$. Cells were recovered from 2 replicate alginate dishes at each time, diluted where necessary and plated out separately and the average taken. Day 0 is approximately 4 h after plating. The dishes were fed with 0·25 ml of DMEM with 15% FCS at 2 weeks as described in previous work with agar (Roscoe & Winslow, 1980).

latent-period cultures. Cells of one such culture, 45F, were therefore recovered from alginate gelled with 40mM and 100mM $CaCl_2$. The results show that recovery of these cells was better from alginate gelled with 100mM $CaCl_2$, and was possible for at least 4 weeks (Table III). In contrast, no viable cells of the control culture, ARBO C9, were recovered after 2 weeks' incubation in alginate gelled with 100mM $CaCl_2$ (Table III). A further experiment was carried out with a second latent-period culture, BE10-7, in which the alginate was gelled with 100mM $CaCl_2$. In view of the results given in Table III, cells were not recovered during the first 2 weeks but the experiment was extended to 6 weeks. The percentage recovery of these cells at Days 0, 14, 28, 42 were 80, 6, 5 and 6·3 respectively. The difference in viability between cells from ENU-exposed latent-period cultures and control lines previously found in agar can therefore be reproduced using alginate and in addition has now been quantified. These results form the basis for longer-term experiments on viable cells and a means of investigating the relationship between prolonged viability and increased fibrinolytic activity. This latter property has also been demonstrated in our latent-period cultures before they acquire the ability to form colonies in agar or tumours in syngeneic rats (Hince & Roscoe, 1978; Roscoe *et al.*, 1980).

The results described here show that growth in alginate can distinguish tumorigenic from non-tumorigenic cells. Alginate also offers the advantage over other media of easy recovery of viable cells over at least 6 weeks. The gelling conditions can be varied in order to attain different objectives. Our results suggest a firmer gel is more conducive to maintenance of viable cells while a less firm gel allows more efficient colony formation. The effect of gel rigidity on colony formation can also be seen in agar. Increasing the agar concentration from 0·3 to 0·5% results in a 20% reduction in PE and a larger proportion of smaller colonies (unpublished observations) as was found with alginate (Table I). It is difficult to establish precisely how much of the added calcium is available physiologically to the cells after cross-linking of the alginate. Although a direct effect of calcium on the cells cannot be ruled out, our data indicate that it does not significantly affect the result. All the cell lines tested showed similar plating efficiencies in agar and alginate (Table II). Furthermore, adding $CaCl_2$ to 0·3% agar in amounts which increased the concentration by 3mM, 9mM and 15mM $CaCl_2$ did not affect the plating efficiency of A15A5 significantly. (The PEs were 43, 54 and 46% respectively compared with a control value of 52%.) These concentrations cover the maximum increase which would be expected (3·6 mM and 9·1 mM) if all the added $CaCl_2$ (40 mM and 100 mM) were available in the alginate layer.

Alginate suspensions should therefore be useful to many workers investigating transformation, anchorage dependence, long-term cell dormancy, clonogenicity of tumour cells and similar phenomena.

We thank Dr D. H. Roscoe for suggesting alginate as a suspending medium and for many helpful discussions. This work has been supported by the Cancer Research Campaign.

### REFERENCES

Barrett, J. C., Crawford, B. D., Mixter, L. O., Schechtman, L. M., Ts'o, P. O. P. & Pollack, R. (1979) Correlation of *in vitro* growth properties and tumorigenicity of Syrian hamster lines. *Cancer Res.*, **39**, 1504.

Courtenay, V. D. & Mills, J. (1978) An *in vitro* assay for human tumours grown in immune-suppressed mice and treated *in vivo* with cytotoxic agents *Br. J. Cancer.* **37**, 261.

Hince, T. A. & Roscoe, J. P. (1978) Fibrinolytic activity of cultured cells derived during ethylnitrosourea-induced carcinogenesis of rat brain. *Br. J. Cancer*, **37**, 424.

Jones, P. A., Laug, W. E., Gardner, A., Nye, C. A., Fink, L. M. & Benedict, W. F. (1976). *In vitro* correlates of transformation in $C_3H/10T_{1/2}$ clone 8 mouse cells. *Cancer Res.*, **36**, 2863.

Lantos, P. L., Roscoe, J. P. & Skidmore, C. J. (1976) Studies of the morphology and tumorigenicity of experimental brain tumours in tissue culture. *Br. J. Exp. Pathol.*, **57**, 95.

MacPherson, I. & Montagnier, L. (1964) Agar suspension culture for the selective assay of cells transformed by polyoma virus. *Virology*, **23**, 291.

Mbanaso, E. N. A. & Roscoe, D. H. (1982). Alginate: an alternative to agar in plant protoplast culture. *Plant. Sci. Letters*, **25**, 61.

Pilwat, G., Washausen, P., Klein, J. & Zimmerman, U. (1980) Immobilisation of human red blood cells. *Z. Naturforsch.*, **35C**, 352.

Roscoe, J. P. (1980) *In vivo–in vitro* analysis of ethylnitrosourea-induced brain carcinogenesis in the rat. *Br. Med. Bull.*, **36**, 33.

Roscoe, J. P. & Claisse, P. J. (1976) A sequential *in vivo–in vitro* study of carcinogenesis induced in the rat brain by ethylnitrosourea. *Nature*, **262**, 314.

Roscoe, J. P. & Claisse, P. J. (1978) Analysis of N-ethyl-N-nitrosourea-induced brain carcinogenesis by sequential culturing during the latent period. I. Morphology and tumorigenicity of the cultured cells and their growth in agar. *J. Natl Cancer Inst.*, **61**, 381.

Roscoe, J. P., Hince, T. A., Claisse, P. J. & Winslow, D. P. (1980) Effect of 12-O-tetradecanoylphorbol-13-acetate on two characteristics of transformation acquired sequentially by by ENU-exposed rat brain cells. *Br. J. Cancer*, **42** 756.

Roscoe, J. P. & Owsianka, A. M. (1982) Alginate: a new reversible gelling medium for investigating cell transformation. *Br. J. Cancer*, **46**, 506.

Roscoe, J. P. & Winslow, D. P. (1980) Increased ability of ethylnitrosourea-exposed brain cells to survive suspension in agar. *Br. J. Cancer*, **41**, 992.

Salmon, S. E., Hamburger, A. W., Soehnlen, B., Durie, B. G. M., Alberts, D. S. & Moon, T. E. (1978) Quantitation of differential sensitivity of human tumor stem cells to anti-cancer drugs. *N. Engl. J. Med.*, **298**, 1321.

Stoker, M. (1968) Abortive transformation by Polyoma virus. *Nature*, **218**, 234.